# A novel malnutrition assessment model predicts the inflammatory storm of relapsed/refractory acute myeloid leukemia following C-type lectin-like molecule-1 chimeric antigen receptor T therapy

**DOI:** 10.3389/fnut.2025.1627624

**Published:** 2025-07-04

**Authors:** Tao Zhang, Mengnan Li, Xiaomei Zhang, Mohan Zhao, Yanyu Jiang, Xin Wang, Yifan Zhao, Xiaoxue Shi, Wentao Qu, Yu Zhang, Xue Bai, Bing Wang, Mingfeng Zhao

**Affiliations:** ^1^Nankai University, Tianjin, China; ^2^The First Central Clinical College of Tianjin Medical University, Tianjin, China; ^3^Department of Hematology, School of Medicine, Tianjin First Central Hospital, Tianjin Thrombosis and Hemostasis Institute, Nankai University, Tianjin, China

**Keywords:** malnutrition, chimeric antigen receptor T (CAR-T), cytokine release storm (CRS), leukeamia, inflammation

## Abstract

**Objectives:**

Previous studies have been insufficient in addressing malnutrition in leukemia patients and inflammatory storms following immunotherapy infusion. This study investigates the relationship between malnutrition and inflammatory storm after C-type lectin-like molecule-1 chimeric antigen receptor T (CLL1 CAR-T) infusion in relapsed/refractory acute myeloid leukemia (r/r AML) patients.

**Methods:**

In this single-center study, we adopted Controlling Nutritional Status (CONUT) and modified Controlling Nutritional Status (mCONUT) to assess the patient’s malnutrition status. The score of CONUT/mCONUT and the severity and grading of cytokine storm at different time points were collected. The area under the receiver operating curve (AUC) was used to evaluate the malnutrition score to predict the early inflammatory storm after CLL1 CAR-T infusion.

**Results:**

Higher malnutrition scores were significantly associated with increased severity of cytokine release storm (CRS). On Day + 7 and Day + 14 after CLL1 CAR-T infusion, the prediction efficiency of the malnutrition assessment model was high, AUC was greater than 0.8, and CONUT Day + 7 reached the peak (AUC = 0.813), and CONUT Day + 14 (AUC = 0.8009). mCONUT Day + 7 reached the peak (AUC = 0.821), and mCONUT Day + 14 (AUC = 0.8162).

**Conclusion:**

Early malnutrition assessment models are practical, objective tools for predicting inflammatory storms in relapsed/refractory AML patients undergoing CLL1 CAR-T therapy.

## Introduction

A high prevalence of malnutrition has been demonstrated in patients with tumors, present in 40%–80% of cases (1). It has been suggested that malnutrition is one of the causes of medical complications and even death (2). Previous studies have primarily focused on the malnutrition of patients with solid tumors, and leukemia patients with worse nutritional conditions were seldom studied. In particular, patients with acute myeloid leukemia (AML) often experience nutritional consumption processes such as hematological stem cell transplantation (HSCT), cellular immunotherapy, chemotherapy, and so on (3). On the other hand, refractory or relapse AML (r/r AML) has a high incidence in the progressive process of AML. Especially when r/r AML is undergoing chimeric antigen receptor (CAR) immunotherapy, the baseline nutritional status may be closely related to the immune response of patients. Because of the uncontrollable inflammatory factor storm or severe infection, r/r AML has a higher mortality rate in the face of malnutrition (4, 5). Therefore, an objective nutritional assessment tool is urgently needed to predict cytokine release storm and immune effector cell-associated neurotoxicity syndrome (CRS/ICANS) during the process of r/r AML accepted CAR-T cell therapy.

At present, it is worth noting that there is no gold standard for the diagnosis or classification of malnutrition. Various nutritional evaluation tools such as body mass index (BMI), weight loss, biochemical indexes, and body composition are applied in different studies (6, 7). The previous study selected patient-generated subjective global assessment (PG-SGA) to evaluate the nutritional status of cancer patients (8). However, PG-SGA usually gets a relatively subjective score, and PG-SGA could not represent the patient’s objective nutritional condition (9). The specificity and sensitivity of the malnutrition universal screening tool (MUST) are relatively low, and nutrition risk screening 2002 (NRS-2002) is relatively not applicable to AML (10). Therefore, we need more objective nutritional evaluation tools to evaluate the nutritional status of patients with r/r AML, and in this case, we could better predict the CRS/ICANS during the process of r/r AML accepted CAR-T cell therapy. Recently, the Controlling Nutritional Status (CONUT) score has been introduced as a nutritional screening tool to recognize the immune response of patients with malignant diseases (11). The CONUT score is an immuno-nutrition screening tool based on serum albumin, total cholesterol, and lymphocyte count. The CONUT score is a potential predicted model to evaluate the nutritional response to CAR-T cell therapy in r/r AML (12).

On the other hand, the human C-type lectin-like molecule 1 (CLL1) usually was highly expressed in leukemia stem cells (LSC) and blast. However, the anti-tumor effect of the CLL1 target also exited the activated pathway of inflammatory cells, influencing processes such as cell adhesion and intercellular signaling crosstalk. In clinics, the tumor microenvironment (TME) of r/r AML has some cytokine storm during CLL1 immunotherapy. These cytokines are derived from monocytes, macrophages, cytotoxic killer T cells, and activated central granulocytes in TME. At the same time, the storm of inflammatory cytokines in TME is closely related to the nutritional status of r/r AML.

Therefore, we select the CONUT score to evaluate the malnutrition of patients with r/r AML and predicted CRS/ICANS of r/r AML patients treated with CLL1 CAR-T by dynamic changes of CONUT.

## Materials and methods

### Patients and study design

A total of 56 patients who underwent CLL1 CAR-T therapy at Tianjin First Central Hospital between May 2019 and May 2025 were retrospectively enrolled. The main inclusion criteria were as follows. (1) AML was diagnosed through the 2008 WHO (World Health Organization) classification of hematological malignancies; Risk classification complies with ELN (European Leukemia Net) classification. (2) R/R (refractory/relapse) status was defined according to the National Comprehensive Cancer Network (NCCN) guidelines; (3) Autologous T cells were used for CAR-T product preparation; (4) medical records within 4 weeks of CAR-T treatment were complete and accessible. The main exclusion criteria were as follows. (1) patients who experienced manufacturing failure of CAR-T products are excluded; (2) incomplete medical records; (3) refusal to sign this informed consent form.

### CLL1 CAR-T therapy

First, the CLL1 CAR-T cell product was an investigational agent manufactured at the Tianjin First Central Hospital. All patients enrolled in this study received CLL1 CAR-T cell infusions following standard manufacturing protocols and inpatient care at our center.

Second, the CLL1 CAR-T construct consisted of a single-chain variable fragment (scFv) targeting CLL1, derived from the FMC63 clone. This scFv was linked to human 4-1BB and CD3 signaling domains and was inserted into the pCDH-MSCV-MCS-EF1-T2A-Pro lentiviral vector. A specific amount of lentivirus was prepared for transduction. Mononuclear cells were isolated from the peripheral blood of patients with acute myeloid leukemia (AML) using a peripheral blood cell separator. CD3-positive T cells were then selected using anti-CD3-coated magnetic beads (Miltenyi, 130-097-043). Subsequently, CD3/CD28-coated magnetic beads (Sigma, 11161-D) and interleukin-2 (IL-2; T&L Biotechnology Co., Ltd.) were added to stimulate T cell expansion. Lentiviral transduction was performed at a multiplicity of infection (MOI) of three after 48 h of T cell expansion.

Third, CLL1 CAR-T cells were infused into patients after 10–15 days of culture, once the activity and number of CLL1 CAR-T cells had stabilized. Prior to CLL1 CAR-T cell infusion, patients underwent lymphodepletion chemotherapy with fludarabine and cyclophosphamide for an average of 3 days, at doses of 25 mg/m^2^/day and 300 mg/m^2^/day, respectively. Additionally, due to high tumor burden, three patients received decitabine and two patients received cytarabine as adjunctive treatment before CLL1 CAR-T cell infusion. The median dose of infused CLL1 CAR-T cells was 1 × 10^7^/kg, with a range of 0.5 × 10^6^–3 × 10^6^/kg.

### Response assessment and follow-up

In total, 56 patients were evaluated 4 weeks after CLL1 CAR-T therapy and according to the response assessment was based on the revised criteria defined by the International Working Group for AML, including: (1) The Eastern Cooperative Oncology Group (ECOG) status score was used to evaluate patient functional status before CAR-T infusion. (2) CRS and ICANS were graded according to the American Society for Transplantation and Cellular Therapy (ASTCT) consensus criteria. The assessment of metabolic and inflammatory parameter mainly includes peripheral blood cell analysis, liver and kidney function, C-reactive protein, ferritin, cytokines, body temperature, blood pressure and oxygen saturation. Physical examination of nervous system (orientation, naming ability, attention, writing ability). Grade 1: Fever (≥ 38°C) without hypotension or hypoxia. Grade 2: Fever with hypotension not requiring vasopressors and/or hypoxia requiring low-flow nasal cannula oxygen (≤ 6 L/min) or blow-by. Grade 3: Fever with hypotension requiring a single vasopressor (with or without vasopressin) and/or hypoxia requiring high-flow nasal cannula oxygen (> 6 L/min), facemask, non-rebreather mask, or venturi mask. Grade 4: Fever with hypotension requiring multiple vasopressors (excluding vasopressin) and/or hypoxia requiring positive pressure ventilation. Mild CRS (Grade 1-2) CRS, and Severe CRS (Grade 3–4). (3) Extramedullary diseases (EMDs) including central nervous system (CNS) leukemia and other systems were defined as AML cells outside the bone marrow (BM), which is detected by cytological, pathological biopsy, or imaging evidence. (4) Complete remission (CR) was defined as normalization of the hemogram and clinical symptoms and marrow with 5% or fewer marrow blasts. (5) Partial remission (PR) refers to clinical symptoms and signs, hemogram, and myelogram, of which 1–2 items are not up to standard. The time points of follow-up are included: 1 day on lymphodepletion (Day BL), 1 day before CLL1 CAR-T cell infusion (Day -1), 3 days after CLL1 CAR-T cell infusion (Day + 3), and 7 days after CLL1 CAR-T cell infusion (Day + 7), 14 days after CLL1 CART cell infusion (Day + 14), and 28 days after CLL1 CAR-T cell infusion (Day + 28). The CONUT scores were accumulated to predict the potential CRS/ICANS rate in r/r AML accepted CLL1 CAR-T immune therapy.

### CONUT score

We utilized the CONUT score and modified the CONUT score (mCONUT). Firstly, The CONUT score evaluates three parameters: serum albumin, total cholesterol, and lymphocyte count. Compared with the CONUT score, the mCONUT score contains C reactive protein (CRP), serum albumin, total cholesterol, and lymphocyte count. The total scores of mCONUT and CONUT are 36 and 24, respectively.

### Statistical analysis

Firstly, the quantitative variables were summarized by mean + standard deviation, median (IQR), and qualitative variables were summarized by absolute frequency and percentage. Secondly, for variables that do not conform to the normal distribution, the non-parametric test was used to compare the differences between the mild CRS group and the severe CRS group. Thirdly, a receiver operating curve (ROC) of the CONUT/mCONUT score cut-off for the most accurate prediction of CRS/ICANS was performed, with an area under the receiver operating curve (AUC) above 0.7 indicating good predictive ability. The *p*-value < 0.05 was considered statistically significant. All the graphs were manufactured with GraphPad Prism 8.0 (GraphPad Software, LLC).

## Results

### Baseline characteristics

This study enrolled 56 patients between May 2019 and May 2025. The median age was 36.5 years (range: 29.25–51), with a gender distribution of 29 males and 27 females. Of the enrolled patients, 21 had primary refractory disease, while 35 cases recurred after previous remission, 14 patients had previous Myelodysplasia Syndrome (MDS) or Myeloproliferative Tumor (MPN), 17 cases were complex karyotypes. According to the ELN classification, 38 cases were high risk, nine cases were medium risk, and nine cases were low risk. 18 patients received previous allo-HSCT, 22 patients with extramedullary disease (EMD), and 25 patients with CNS leukemia. According to the ECOG performance status, 24 patients scored < 2 and 32 patients scored ≥ 2. Median albumin level was 36.95 g/L, ranging from 34.71 to 42.73 g/L. The median of total cholesterol was 3.69 mmol/L, ranging from 2.94 to 4.46 mmol/L. The median lymphocyte count was 0.67 × 10^9^/L, ranging from 0.4 × 10^9^ to 1.08 × 10^9^/L. The median of C reactive protein (CRP) was 10.53 mg/L, ranging from 2.49 to 44.77 mg/L. The median tumor burden was 37.5%, ranging from 11% to 72.5%. The median CLL1 (+) rate of tumor cells in patients was 89%, ranging from 77.5% to 95%. The median dose of CLL1 CAR-T cells administered was 1 × 10^7^/kg, ranging from 0.5 to 3 × 10^6^/kg. (Table 1).

**TABLE 1:** The baseline data of enrolled patients.

Characteristics	Total (*N* = 56)
Age median (range)	36.5 (29.25–51.0)
Sex male/female (*n*)	29/27
Body mass index, mean (SD)	22.51 (4.34)
Primary refractory disease (*n*)	21
Relapsed disease (*n*)	35
MDS/MPN history (*n*)	14
Complex karyotypes	17
**ELN classification (*n*)**	
High risk	38
Intermediate risk	9
Low risk	9
**Previous chemotherapy regimens**	
Cytarabine + anthracyclines (IA/DA/HA)	56
High/intermediate-dose cytarabine	11
FLAG/CLAG	16
CAG/HAG	35
Venetoclax + Chemotherapy (*n*)	31
Other targeted drugs (giltertinib, sorafenib) (*n*)	9
Previous allo-HSCT (*n*)	18
EMD (n)	22
CNS leukaemia (*n*)	25
**ECOG score**	
< 2	24
≥ 2	32
Tumor burden before CAR-T infusion median (range, %)	37.5 (11–72.5)
CLL1 expression median (range, %)	89 (77.5–95)
CLL1 CAR-T infusion dose median (range, %)	1 (0.5–3)
Bridge HSCT (*n*)	28
**Baseline COUNT/mCOUNT**	
Albumin median (range)	36.95 (34.71–42.73)
Total cholesterol median (range)	3.69 (2.94–4.46)
Lymphocyte count median (range)	0.67 (0.4–1.08)
CRP median (range)	10.53 (2.49–44.77)

MDS, myelodysplastic syndromes; MPN, myeloproliferative neoplasm; HSCT, hematopoietic stem cell transplantation; CLL-1, C-type lectin-like molecule 1; CNS, central nervous system; CONUT, Controlling Nutritional Status; mCONUT, modified Controlling Nutritional Status; EMD, extramedullary disease; ELN, European Leukemia Net; ECOG, Eastern Cooperative Oncology Group, albumin (g/L), total cholesterol (mmol/L), lymphocyte count (10^9^/L); CRP, C creative protein (mg/L).

### Therapy response to CLL1 CAR-T

The efficacy evaluation was conducted on 56 patients within 4 weeks, excluding one patient who experienced cerebral hemorrhage following CAR-T cell infusion. The overall remission rate was 73.2% (41/56), with 28 patients achieving complete remission (CR) and 13 patients with partial remission (PR). A total of 15 patients had no response (NR). The median of overall survival (OS) is 8 months, ranging from 1 to 21.75 months. The median of progression-free survival (PFS) is 5 months, ranging from 1 to 14.5 months. One day before lymphodepletion (Day BL), and CONUT score to predict the therapeutic response of CLL1 CAR-T. Compared with 28 CR patients, 15 NR patients had higher CONUT scores, and the *P*-value was 0.0121. In addition, compared with 13 PR patients, 15 NR patients also had higher CONUT scores, and the *P*-value was 0.0081.

### Adverse response during CAR-T cell therapy

**FIGURE 1:**
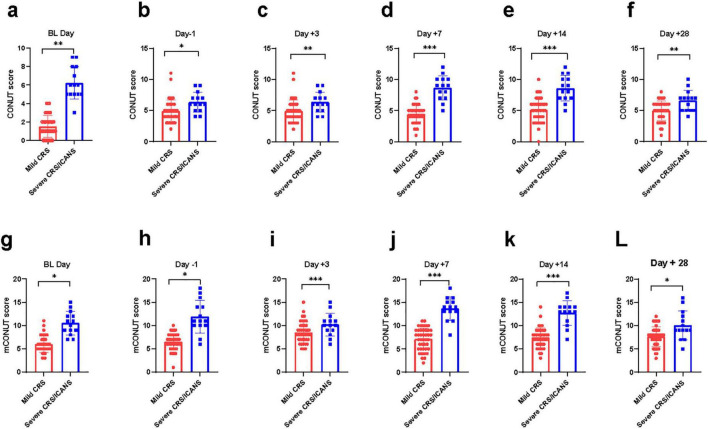
A comparison of CRS/ICANS at 21differing time points is hereby presented. The difference of CONUT/mCONUT score between two group at various time points. Mann-Whitney Test. **(a, g)** day on lymphodepletion, **(b, h)** 1 day before CLL1 CAR-T cell infusion, **(c, i)** 3 days after CLL1 CAR-T cell infusion, **(d, j)** 7 days after CLL1 CAR-T cell infusion, **(e, k)** 14 days after CLL1 CART cell infusion, **(f, l)** 28 days after CLL1 CAR-T cell infusion. *P < 0.05, **P < 0.05, ***P < 0.005.

Among the 56 enrolled patients, 42 patients had grade 1/2 CRS, and 14 patients had 3/4 grade CRS. In patients with severe inflammatory factor storm, 5/14 of them had ICANS, four patients had 1/2 grade ICANS, and one patient had 3/4 grade ICANS (Figure 1). The median onset of CRS occurred on Day + 8, ranging from Day + 5 to Day + 16; and the peak severity of CRS was observed on Day + 7, ranging from Day + 5 to +12. Corticosteroids and tocilizumab were used to control CRS or ICANS syndrome in 37 and 19 patients, respectively. There was no significant correlation between the severity of CRS/ICANS and the therapeutic effect. In addition, hemocytopenia is the most common adverse event. 47 of 56 patients had grade 3/4 agranulocytosis. However, it is worth noting that 28, 25, and 19 patients developed agranulocytosis, moderate anemia, and thrombocytopenia before CAR-T treatment because the primary disease was not cured. The patients experienced bacterial, fungal, and viral infections (19 cases of bacterial infection, 14 cases of fungal infection, eight cases of viral infection) and were relieved after receiving antibiotic treatment, and the treatment was based on pathogenic microorganism blood culture and NGS sequencing.

### Dynamic change of CONUT

**FIGURE 2:**
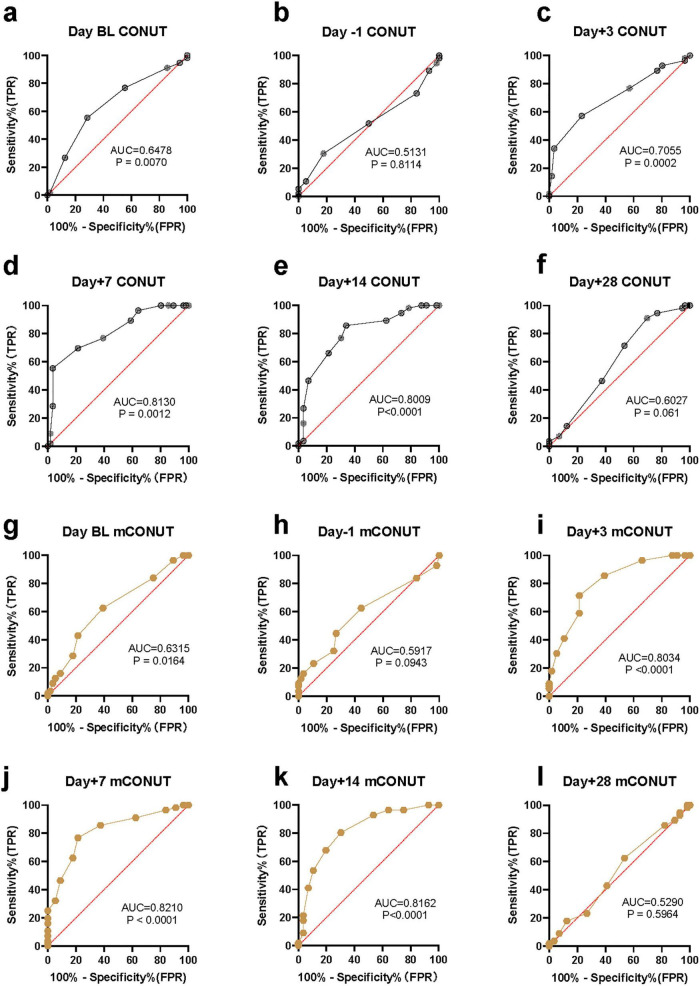
The statistical results of malnutrition score in predicting an inflammatory storm. ROC curve and AUC in predictive value. Wilson-Brown test. **(a–f)** The predictive efficiency of CONUT assessment model. **(g–l)** The predictive efficiency of mCONUT assessment model.

The results showed that at the two cut-off points before CAR-T infusion, the efficiency of CONUT in predicting CRS was relatively low. They were BL day (AUC = 0.6478, *P* = 0.007) and Day -1 (AUC = 0.5131, *P* = 0.8114). On the third day after CAR-T infusion, the prediction efficiency of CONUT gradually increased, Day + 3 (AUC = 0.7055, *P* = 0.0002). On the 7*^th^* and 14*^th^* day after CAR-T infusion, the prediction efficiency of CONUT increased significantly, AUC was greater than 0.8, Day + 7 reached the peak (AUC = 0.813, *p* < 0.0001), and Day + 14 (AUC = 0.8009, *p* < 0.0001). However, on the 28*^th^* day after CAR-T infusion, the prediction value of CONUT was not statistically significant, Day + 28 (AUC = 0.6027, *P* = 0.061) (Figure 2).

### Dynamic change of mCONUT

The results showed that at the two critical points before CAR-T infusion, the statistical prediction efficiency of CONUT and mCONUT were consistent, and the efficiency of predicting CRS was low. On Day BL (AUC = 0.6315, *P* = 0.6315) and Day -1 (AUC = 0.5917, *P* = 0.5917), respectively. However, on the third day after CAR-T infusion, compared with the prediction efficiency of CONUT, the prediction efficiency of mCONUT was higher, Day + 3 (AUC = 0.8034, *p* < 0.0001). On the 7*^th^* and 14*^th^* days after CAR-T infusion, the prediction efficiency of mCONUT was also significantly higher than that of CONUT, and reached the peak on Day + 7 (AUC = 0.821, *p* < 0.0001), Day + 14 (AUC = 0.8162, *p* < 0.0001). However, 28 days after CAR-T infusion, the predictive value of mCONUT was not statistically significant (AUC = 0.529, *P* = 0.5964) (Figure 2).

### The predictive efficiency of CONUT/mCONUT

On Day + 7 and Day + 14 after CLL1 CAR-T infusion, the prediction efficiency of the malnutrition assessment model was high, AUC was greater than 0.8, and CONUT Day + 7 reached the peak (AUC = 0.813), and CONUT Day + 14 (AUC = 0.8009). mCONUT Day + 7 reached the peak (AUC = 0.821), and mCONUT Day + 14 (AUC = 0.8162). The predictive efficiency of mCONUT is higher than CONUT.

## Discussion

Chimeric antigen receptor T-cell therapy, an adoptive immunotherapy, expresses one or more specific chimeric antigen receptors on T cells through genetic engineering, which can target tumor cells. CAR-T is a milestone in hematological immunotherapy in recent years. In recent years, more and more targets have been used in preclinical study, such as CLL1, CD33, CD123, CD47, CD70, siglec6, CD93, CD64, and TIM3 (13). Our study demonstrated that the malnutrition status of AML patients was associated with CRS in patients, who accepted CLL1 CAR-T infusion, and the dynamic assessment of malnutrition status could be used to predict CRS. In this case, we select the CONUT score and modified CONUT (mCONUT) to foresee CRS/ICANS after CART infusion. Meanwhile, this study demonstrates that the malnutrition status of patients with r/r AML, as assessed by the CONUT and mCONUT scores, is significantly associated with the occurrence and severity of CRS/ICANS following CLL1 CAR-T therapy. The dynamic changes in these scores provide valuable predictive insights, particularly at key time points such as Day + 7 and Day + 14 post-infusion, where the AUC values for CONUT and mCONUT scores were notably high (AUC > 0.8) (Figure 2). These findings underscore the importance of malnutrition assessment in managing CAR-T-related CRS.

Firstly, the CONUT score, which incorporates serum albumin, total cholesterol, and lymphocyte count, reflects both nutritional and immune status. Our results indicate that patients with higher baseline CONUT scores were more likely to experience severe CRS/ICANS, suggesting that malnutrition may exacerbate inflammatory responses. This aligns with previous studies highlighting the role of malnutrition in impairing immune function and increasing susceptibility to cytokine storms (14). A previous study has shown that hypoalbuminemia is a highly sensitive marker to predict the immune response in tumor patients. Albumin serves as the carrier of acquired immune antibodies, and hypoalbuminemia is often associated with immune dysfunction (15). The invasion of opportunistic pathogenic microorganisms caused by hypoalbuminemia can stimulate the release of additional cytokines through a positive feedback loop (16). On the other hand, under physiological conditions, the physiological level of total cholesterol (TC) can maintain the structure of the cell membrane signaling pathway, which could maintain the function of immune cells (17). When malnutrition occurs, hypocholesterolemia can further impair immune cell homeostasis and indirectly promote the release of cytokines (18). At the same time, hypocholesterolemia can exacerbate the tumor microenvironment and promote tumor cells to release more intracellular fatty acids and cytokines, such as IL-4(Interleukin-4), arachidonic acid, etc., (19). It is worth noting that when malnutrition occurs, the lymphocyte count is even lower, which is often linked to fatal septic shock and directly accelerates the release of cytokines (20, 21). Therefore, malnutrition is closely related to cytokine storm after adoptive immunotherapy.

Secondly, CRP in the mCONUT score further enhanced its predictive accuracy, as CRP is a well-established marker of systemic inflammation. The significant differences in mCONUT scores between mild and severe CRS/ICANS groups support its utility in risk stratification. In our study, patients with higher malnutrition scores had higher levels of inflammatory markers, such as CRP, IL-6 (Interleukin-6), and IL-10 (Interleukin-10), reinforcing the link between nutritional status and cytokine dysregulation. The release of IL-6 and CRP typically follows a positive feedback mechanism, and the positive feedback release of IL-6 may be the most significant in malnutrition after CLL1 CAR-T infusion, which is closely related to the activation of cellular immunotherapy assisted by IL-6 (22).

Notably, the dynamic changes in CONUT and mCONUT scores post-infusion were correlated with the timing of CRS onset and peak severity, suggesting that these scores could serve as early warning tools for clinicians to intervene proactively. Then, the ROC analysis further validated the predictive power of CONUT and mCONUT scores, with the highest AUC values observed at Day + 7 and Day + 14. These time points coincide with the typical peak of CRS, indicating that nutritional assessment during this window is critical for predicting severe CRS. However, the decline in predictive accuracy by Day + 28 may reflect the resolution of acute inflammatory responses, emphasizing the importance of early monitoring. Therefore, early assessment of malnutrition, including hypoalbuminemia, hypocholesterolemia, low lymphocyte counts, and CRP, is more conducive to early prediction of inflammatory factor storm after CAR-T therapy in r/r AML patients.

**FIGURE 3:**
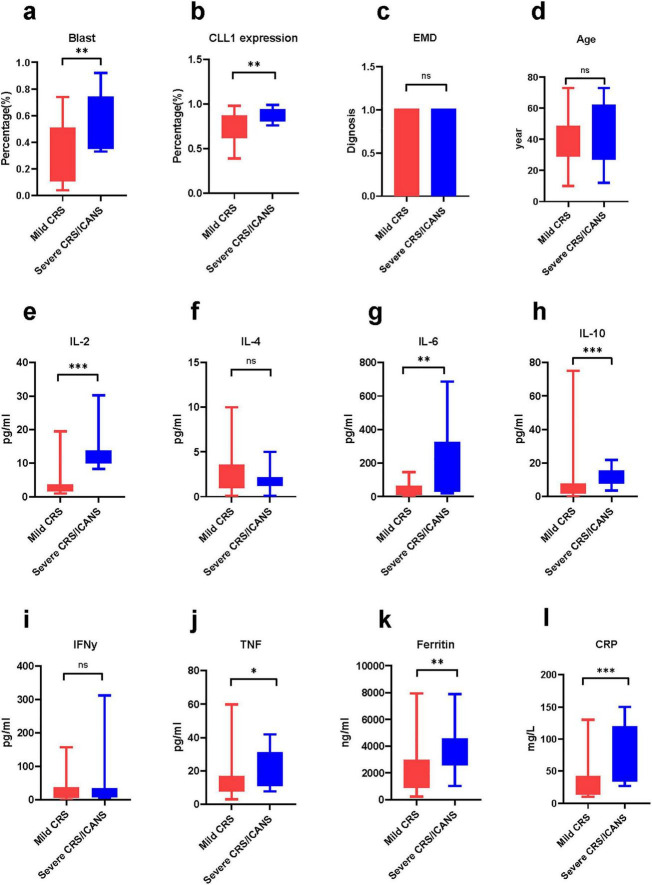
The difference in cytokines between the mild group and the severe group. The comparison of various cytokines and baseline condition between two group. Mann-Whitney Test. **(a)** Blast, **(b)** CLL1 expression, **(c)** extramedullary disease, **(d)** age **(e)** Interleukin-2, **(f)** Interleukin-4, **(g)** Interleukin-6, **(h)** Interleukin-10, **(i)** interferon-γ, **(j)** tumor necrosis factor, **(k)** ferritin, **(l)** C creative protein *P < 0.05, **P < 0.05, ***P < 0.005.

**FIGURE 4:**
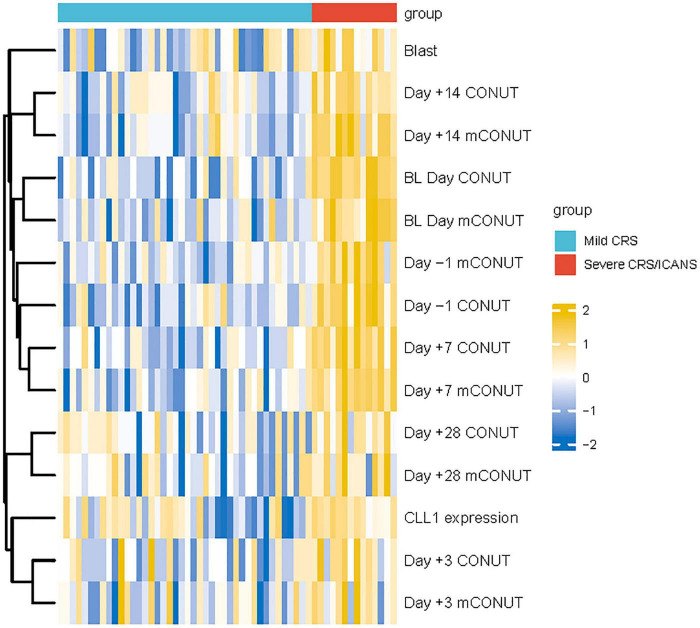
The heat map summarizes the differences between the mild and severe groups.

Notably, the baseline CONUT and mCONUT scores, along with tumor burden (Blast percentage), are independent predictors of severe CRS/ICANS (Figures 3, 4). In fact, in the tumor microenvironment, the higher the blast percentage, the more inflammatory factors released by tumor cells, such as IL-10, TGF (tumor growth factor), and further promote the recruitment of chemokines and build a platform for inflammatory storms (23). At the same time, the expression of CLL1 on leukemia stem cells was also positively correlated with the release of cytokines, which may be related to the killing effect (24). These two correlations are consistent with previous research conclusions. However, the severity of CRS/ICANS is not related to extramedullary invasion, age, IL-4, IFN -γ(interferon-γ), etc., This underscores the multifactorial nature of CRS/ICANS and the need for comprehensive risk assessment. A previous study in our center showed no significant difference in the treatment effect of CLL1 CAR-T in patients with extramedullary infiltration and non-extramedullary infiltration. This is consistent with our previous study (25). Moreover, IL-4 mainly comes from T helper cell subsets, mast cells, and basic granulocytes. Most of them are involved in allergic reactions. There was no significant difference between mild and severe CRS groups. We are also eager for future studies to further confirm.

In conclusion, the CONUT and mCONUT scores are practical, objective tools for predicting CRS/ICANS in r/r AML patients undergoing CLL1 CAR-T therapy. Integrating these scores into clinical practice could enhance risk stratification, guide early interventions, and improve patient outcomes. As CAR-T therapy evolves, optimizing baseline patient conditions, including nutritional status, will be pivotal in minimizing toxicity and maximizing efficacy.

### Future and limitation

The successful application of targeted CLL1 monoclonal antibodies to AML cells exemplifies the promising future of CLL1 targeting in immunotherapy. In this case, CLL1 chimeric antigen receptor-T (CAR-T) cell therapy has become a new treatment option for most r/r AML. Meanwhile, we anticipate further CAR modifications aimed at reducing the inflammatory factor storm without compromising the curative effect. In terms of limitations, first, this study was a single-center study. Second, the sample size included in this study was relatively small. We sincerely look forward to larger-scale, multi-center studies to further explore the relationship between malnutrition and the inflammatory storm.

## Data Availability

The datasets presented in this article are not readily available because, the dataset of this article covers the privacy of patients treated with CAR-T therapy. Requests to access the datasets should be directed to MiZ.
